# Relationship between obesity and asthma symptoms among children in Ahvaz, Iran: a cross sectional study

**DOI:** 10.1186/1824-7288-37-1

**Published:** 2011-01-06

**Authors:** Tahereh Ziaei Kajbaf, Shideh Asar, Mohammad Reza Alipoor

**Affiliations:** 1 Pediatric Department, Abuzar Children's Hospital, Ahvaz Jondishapour University of Medical Sciences, (Golestan street), Ahvaz, Iran; 2 Pediatric Department, Golestan Children's Hospital, Ahvaz Jondishapour University of Medical Sciences, (Golestan street), Ahvaz, Iran; 3 Pediatric Cardiology Department, Iran University of Medical Sciences,(valiasr street)Tehran, Iran

## Abstract

**Background:**

Obesity has been identified as a risk factor for higher prevalence of asthma and asthma-related symptoms in children. The objective of this study was to evaluate the relationship between the prevalence of asthma symptoms and obesity among school-age children in the city of Ahvaz, Iran.

**Methods:**

A total of 903 children, 7 to 11 years of age, were enrolled in this study through cluster sampling. The International Study of Asthma and Allergies in Childhood (ISAAC) questionnaire was used to identify the children who were currently suffering from asthma. Height and weight were measured and body mass index (BMI) was calculated in kg/m^2^. Overweight was defined as BMI greater than the age- and sex-specific 85th percentile, and obesity as BMI greater than the 95th percentile. We determined the relationship between obesity and asthma symptoms by chi-square tests.

**Results:**

The prevalence of wheeze ever, current wheezing, obesity, and overweight was 21.56%, 8.7%, 6.87%, and 9.5%, respectively. The current prevalence of wheezing among obese and overweight children was 68.75% and 37%, respectively, and there was a statistical association between obesity and the prevalence of current wheezing (p < 0.001), night cough (p < 0.001), and exercise-induced wheezing (p = 0.009), but obesity and overweight were not associated with eczema and allergic rhinoconjunctivitis, so it seems that the pathophysiology of asthma in obese and overweight children is not related to allergy.

**Conclusion:**

There is a strong association between asthma symptoms and both overweight and obesity in both sexes among school-age children.

## Introduction

Asthma is characterized by episodic airflow obstruction, increased airway responsiveness, and airway inflammation. Asthma is a growing threat to the health of children [1]. The prevalence of asthma among school-age children has been rising in many regions of the developed world [2-6]. Also, in the past 2 decades there has been a significant increase in the prevalence of obesity among children worldwide [7].

It has been recognized that obesity is more common among children with asthma, and associations between asthma and obesity have been observed in cross-sectional studies of adults and children [8,9]. Prospective studies with healthy non-asthmatic participants showed that a gain in weight increased the risk of asthma in adults and adolescents [10,11]. Finally, some studies in asthmatic patients showed that weight loss resulted in an improvement in lung function, symptoms, and health status [12,13]. Nevertheless, the relationship between obesity and asthma remains controversial [14] as some studies could not disclose any significant correlation between asthma as defined by bronchial hyper-responsiveness and obesity, and explained that the reported association between asthma and obesity might be due to a misinterpretation of noisy breathing in overweight subjects [15,16]. The obesity is a potentially modifiable risk factor, in which its relationship to asthma incidence should be clarified [17].

This study was conducted to determine whether obesity, as measured by body mass index (BMI), was associated with a higher prevalence of asthma symptoms. Possible differences between boys and girls were also evaluated.

## Methods

In the autumn of 2009, we studied a random sample of children in the city of Ahvaz, Iran. The sampling frame included primary schools that were randomly selected from different parts of Ahvaz to obtain the required number of children. All children aged 7 to 11 years at each selected school were invited to participate and were studied after their parents' informed consent was obtained. The International Study of Asthma and Allergies in Childhood (ISAAC) questionnaire [18] was used to identify the children currently suffering from asthma. Standard guidelines for translation of the questionnaire from English into the Persian was used [19]. The questionnaires were distributed to parents of 1000 children. The questionnaire included questions on demographic characteristics, wheeze ever, current history of wheezing, diagnosed asthma, exercise-induced wheezing, and nocturnal cough. The questionnaire was completed at the schools by the children's parents and researchers. In this study, wheeze ever is defined as any history of wheezy breathing; current wheezing is a history of at least 1 attack of wheezing during the last 12 months; diagnosed asthma is the same as wheeze ever plus a doctor's diagnosis of asthma.

Allergic rhinitis was diagnosed by enquiring with questions such as history of sneezing or blocked nose during the last 12 months when he/she did not have a cold or flu and has your child ever had hay fever? Questions concerning the diagnosis of eczema were as follows: has your child ever had a recurrent itchy rash for at least 6 months and has your child ever had eczema.

The height and weight of all the children were measured. BMI was calculated by dividing weight in kg by the square of the height in meters (kg/m^2^) [20]. The BMI was found to be the single best predictor of body fatness in children [21]. The BMI values of the patients in the study were compared with the international cut-off points per age and sex provided by Cole et al [22]. Overweight was defined as BMI of the age- and sex-specific >85^th ^to <95^th ^percentile, and obesity as BMI greater than the 95^th ^percentile [23]. The sample size was 750, and was calculated using the following formula:

$$n=\frac{z^{2}\times p(1-p)}{d^{2}}$$

In our study, d = 0.012, z = 1.96, and p = 0.028 (according to the prevalence of asthma symptoms and obesity among school children in other studies) [24]. The results were analyzed statistically by applying the Student's t-test and the chi-square test; p < 0.05 was considered significant.

## Results

Parents of 903 children responded to the questionnaire (response rate, 90.3%). Approximately 34% of the children were male and 66% were female. The median age was 8.95 years. The lifetime prevalence of wheezing (wheeze ever) was 21.56%. The current prevalence of wheezing was 8.7% and of doctor-diagnosed asthma was 2% (Table 1).

**Table 1:** Prevalence of asthma symptoms among school-age children

Symptoms	**No**.	%
Wheeze ever	194	21.56
Wheeze in the past year	79	8.7
Doctor-diagnosed asthma ever	17	2
Night cough	65	7.2
Exercise-induced wheeze	31	3.4

In this study, prevalence of current wheezing during the past 12 months decreased with increasing age (p < 0.02) (Table 2), and current prevalence of wheezing was higher among boys than girls (12% vs. 7%, respectively; p = 0.012) (Table 3). The prevalence of obesity and overweight among the children was 6.87% and 9.5%, respectively. The prevalence of obesity and overweight among girls and boys was found to be different (Table 4).

**Table 2:** Prevalence of wheeze in the past year in different ages

Age (yrs)	**No**.	Wheeze in the Past Year	
		
		**No**.	%
7	193	25	13
8	180	20	11.1
9	183	15	8.2
10	172	11	6.4
11	175	8	4.6

**Table 3:** Prevalence of wheeze in the past year with significant difference in the both sexes

Wheeze in the Past Year	Boy	Girl	Total
	
	N = 308	N = 595	N = 903
	**No. (%)**	**No. (%)**	**No. (%)**
	
12-month prevalence of wheezing	37 (12)	42 (7)	79 (8.7)

**Table 4:** Prevalence of obesity and overweight in school-age children by sex

Sex	Prevalence		%	
	
	Overweight		Obesity	
	**No**.	**%**	**No**.	**%**

Both	86	9.5	62	6.87
Boys	34	11	29	9.4
Girls	52	8.73	33	5.54

The current prevalence of wheezing among obese children was 68.75% and among overweight children was 37%, and there was a statistical association between prevalence of current wheezing and obesity (p < 0.001). Current wheezing during the past 12 months increased with increasing weight (Table 5).

**Table 5:** Prevalence of current wheezing and exercise-induced wheezing among obese, overweight and normal weight school-age children

Symptoms	Obesity N = 64		Overweight N = 84		Normal weight N = 755		p value
	**No**.	**%**	**No**.	**%**	**No**	**%**	
	
Wheeze in the past year	44	68.75	31	37	4	0.53	<0.001
Exercise-induced wheeze	15	23.4	13	15.5	3	0.4	0.009

There was no statistical association between obese and overweight children with current wheeze in either sex (p = 0.18).

**Figure 1:**
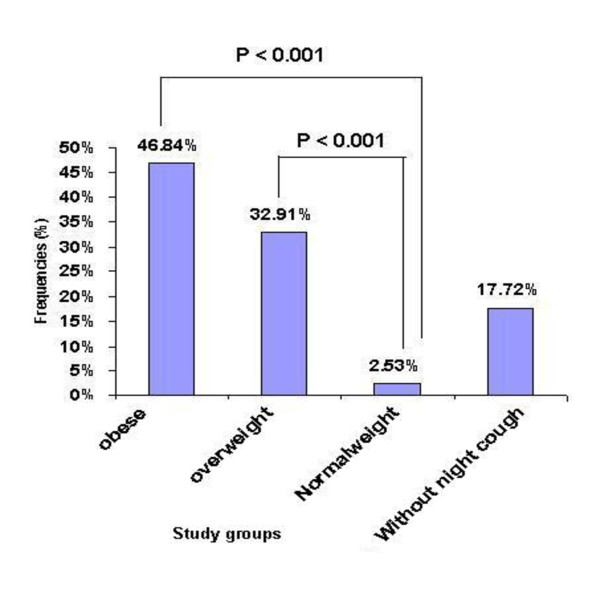
**Comparing the frequencies of night cough in obese, overweight, and normal weight children with current wheeze to without night cough children**.

The frequencies of night cough among obese and overweight children with current wheezing was 37(46.84%) and 26 (32.91%) respectively, but among normal-weight children with current wheezing it was 2 (2.53%) (p < 0.001, P < 0.001, respectively) followed by 14 (17.72%) cases without night cough (Figure 1). The prevalence of night cough was higher in obese and overweight children than in normal-weight children (p < 0.001).

Our analysis showed that 23.4% of obese children and 15.5% of overweight children had current symptoms of exercise-induced wheeze, but only 0.4% of non-obese children had current symptoms of exercise-induced wheeze (p = 0.009) (Table 5).

Our study demonstrated that 4.6% of obese children and 5% of overweight and normal-weight children had a history of allergic rhinoconjunctivitis, and the prevalence of eczema among obese, overweight, and normal-weight children was 1.5%, 2.3%, and 2.6%, respectively. There was no significant statistical association between obese, overweight, and normal-weight children in eczema and allergic rhinoconjunctivitis (p > 0.05).

## Discussion

This study showed that obese children had significantly higher prevalence of current wheezing than children of normal weight (68.7% vs. 0.53%). Also, the prevalence of current wheezing among overweight children was higher than in normal-weight children (37% vs. 0.53%). These associations are consistent with other studies [11,25-32].

Our findings demonstrated an increased prevalence of exercise-induced wheezing among overweight and obese children compared with that in normal-weight children. Obese children had 58 times the prevalence of exercise-induced wheezing than normal-weight children (23.4% vs. 0.4%), and there was 38 times the prevalence of exercise-induced wheezing among overweight children in comparison with normal-weight children (15.5% vs. 0.4%). Our findings are consistent with other studies [33,34].

Exertional wheeze in overweight and obese subjects may be due to an increase in the work of breathing, with upper airway collapse or changes in lung mechanics increasing the load on the upper airways. Other proposed mechanisms of exercise-induced wheezing include: 1) mucosal drying and increased osmolality stimulating mast cell degranulation, and 2) rapid airway rewarming after exercise causing vascular congestion, increased permeability, and edema leading to obstruction [35].

In our study, the relationship between current wheezing and obesity and overweight was independent of sex (p = 0.18). This finding was consistent with reports by others [26,30,36-38], but some reports have noted the association only in women, or that it was stronger in women [8,11,39,40]. This inconsistency in results for boys and girls between studies may be attributed to differences in study populations, the age distribution of participants, and different definitions of asthma used.

Despite a strong association of obesity with current wheezing and exercise-induced wheezing, no association was found between obesity and allergic rhinoconjunctivitis and eczema. This contradicts results from some studies [41,42], but is consistent with results from some other studies [27,43].

If one assumes that subjects without a history of allergy are less likely to have allergic asthma, our observations suggest that excessive symptoms of current wheezing and exercise-induced wheezing among overweight and obese children may be due to causes other than allergy. Obesity and overweight are associated with an increase in the occurrence of both gastro-esophageal reflux and sleep apnea [44], and both of these conditions may mimic the symptoms of wheeze or cough without changes in lung function or airway responsiveness [45].

On the basis of these findings, overweight and obesity seem to be significant risk factors for current wheezing and exercise-induced wheezing; if these can be considered to be modifiable risk factors, interventions that affect weight loss could be associated with a decrease in asthma symptoms. Thus, children with co-morbid asthma and obesity should be encouraged to increase physical activity and lose weight.

There are several limitations in this study because it is a cross-sectional study; the data did not provide direct information on whether obesity preceded the development of asthma, which has been discussed in previous studies [10,11]. A measurement of airway responsiveness is desirable but not practical in studies like ours, and we did not have objective measures for the allergic status.

In conclusion, the results of this study suggest that in our region there is a strong association between asthma symptoms and both overweight and obesity in both sexes among school-age children.

## Competing interests

The authors declare that they have no competing interests.

## Authors' contributions

All authors contributed to all aspects, including searching the literature and writing the article. All authors have read and approved the final manuscript.

## References

[B1] Redd SC Asthma in the United States burden and current theories Environ Health Perspect 2002 110 4 557 60 12194886 10.1289/ehp.02110s4557 PMC1241205

[B2] Asher MI Barry D Clayton T Crane J D'Souza W Ellwood P Ford RP Mackay R Mitchell EA Moyes C Pattemore P Pearce N Stewart AW International Study of Asthma and Allergies in Childhood (ISAAC) Phase One. The burden of symptoms of asthma, allergic rhinoconjunctivitis and atopic eczema in children and adolescents in six New Zealand centres: ISAAC Phase One N Z Med J 2001 114 1128 114 20 11346157

[B3] ISAAC Steering Committee Worldwide variation in prevalence of symptoms of asthma, allergic rhinoconjunctivitis, and atopic eczema: ISAAC. The International Study of Asthma and Allergies in Childhood (ISAAC) Steering Committee Lancet 1998 351 1225 32 10.1016/S0140-6736(97)07302-9 9643741

[B4] Mannino DM Homa DM Redd SC Involuntary smoking and asthma severity in children: data from the third National Health and Nutrition Examination survey Chest 2002 122 409 15 10.1378/chest.122.2.409 12171810

[B5] Pekkanen J Xu B Jarvelin MR Gestational age and occurrence of atopy at age 31: a prospective birth cohort study in Finland Clin Exp Allergy 2001 31 95 102 11167956

[B6] Woolcock AJ Peat JK Evidence for the increase in asthma worldwide Ciba Found Symp 1997 206 122 34 9257009 10.1002/9780470515334.ch8

[B7] Mokdad AH Serdula MK Dietz WH Bowman BA Marks JS Koplan JP The spread of the obesity epidemic in the United States, 1991-1998 JAMA 1999 282 16 1519 22 10.1001/jama.282.16.1519 10546690

[B8] Figueroa-Munoz J Chinn S Rona R Association between obesity and asthma in 4-11 year old children in the UK Thorax 2001 56 133 7 10.1136/thorax.56.2.133 11209102 PMC1745999

[B9] Moudgil H Prevalence of obesity in asthmatic adults BMJ 2000 321 7258 448 10.1136/bmj.321.7258.448 10991591 PMC1127809

[B10] Camargo CA Jr Weiss ST Zhang S Willett WC Speizer FE Prospective study of body mass index, weight change, and risk of adult-onset asthma in women Arch Intern Med 1999 159 21 2582 8 10.1001/archinte.159.21.2582 10573048

[B11] Castro-Rodríguez JA Holberg CJ Morgan WJ Wright AL Martinez FD Increased incidence of asthmalike symptoms in girls who become overweight or obese during the school years Am J Respir Crit Care Med 2001 163 6 1344 9 11371399 10.1164/ajrccm.163.6.2006140

[B12] Hakala K Stenius-Aarniala B Sovijarvi A Effects of weight loss on peak flow variability, airways obstruction, and lung volumes in obese patients with asthma Chest 2000 118 1315 21 10.1378/chest.118.5.1315 11083680

[B13] Stenius-Aarniala B Poussa T Kvarnström J Grönlund EL Ylikahri M Mustajoki P Immediate and long term effects of weight reduction in obese people with asthma: randomised controlled study BMJ 2000 320 7238 827 32 10.1136/bmj.320.7238.827 10731173 PMC27319

[B14] Redd SC Mokdad AH Invited commentary: obesity and asthma: new perspectives, research needs, and implications for control programs Am J Epidemiol 2002 155 198 202 10.1093/aje/155.3.198 11821242

[B15] Shaheen SO Sterne JA Montgomery SM Azima H Birth weight, body mass index and asthma in young adults Thorax 1999 54 396 402 10.1136/thx.54.5.396 10212102 PMC1763790

[B16] Wickens K Barry D Friezema A Rhodius R Bone N Purdie G Crane J Obesity and asthma in 11-12 year old New Zealand children in 1989 and 2000 Thorax 2005 60 1 7 12 10.1136/thx.2002.001529 15618575 PMC1747164

[B17] Beuther DA Sutherland ER Overweight, obesity, and incident asthma: a meta-analysis of prospective epidemiologic studies Am J Respir Crit Care Med 2007 175 7 661 6 Epub 2007 Jan 18. [PMID: 17234901] 10.1164/rccm.200611-1717OC 17234901 PMC1899288

[B18] Asher MI Keil U Anderson HR Beasley R Crane J Martinez F Mitchell EA Pearce N Sibbald B Stewart AW Strachan D Weiland SK Williams HC International Study of Asthma and Allergies in Childhood (ISAAC): rationale and methods Eur Respir J 1995 3 483 91 10.1183/09031936.95.08030483 7789502

[B19] Weiland SK Beasley R Strachan D Guidelines for the translation of questionnaires 1993 Münster Germany: ISAAC phase one coordinating committee

[B20] Daniels SR Khoury PR Morrison JA The utility of body mass index as a measure of body fatness in children and adolescents: differences by race and gender Pediatrics 1997 99 804 7 10.1542/peds.99.6.804 9164773

[B21] Roche AF Siervogel RM Chumlea C Webb P Grading body fatness from limited anthropometric data Am J Clin Nutr 1981 34 2831 8 7315784 10.1093/ajcn/34.12.2831

[B22] Cole TJ Bellizzi MC Flegal KM Dietz WH Establishing a standard definition for child overweight and obesity worldwide: international survey BMJ 2000 320 7244 1240 10.1136/bmj.320.7244.1240 10797032 PMC27365

[B23] Aggarwal T Bhatia RC Singh D Sobti PC Prevalence of obesity and overweight in affluent adolescents from Ludhiana, Punjab Indian Pediatr 2008 45 500 2 18599939

[B24] Mirsaeid Ghazi B Sharifi SH Goodarzipoor K Aghamohammadi A Atarod L Rezaei N Kouhi A The Prevalence of Asthma among the Students (7-18 Years Old) in Tehran during 2002-2003 Iran J Allergy Asthma Immunol 2004 3 2 89 92 17301398

[B25] Gilliland FD Berhane K Islam T McConnell R Gauderman WJ Gilliland SS Avol E Peters JM Obesity and the risk of newly diagnosed asthma in school-age children Am J Epidemiol 2003 158 5 406 15 10.1093/aje/kwg175 12936895

[B26] Beuther DA Sutherland ER Overweight, obesity, and incident asthma: a meta-analysis of prospective epidemiologic studies Am J Respir Crit Care Med 2007 175 661 6 10.1164/rccm.200611-1717OC 17234901 PMC1899288

[B27] Schachter LM Salome CM Peat JK Woolcock AJ Obesity is a risk for asthma and wheeze but not airway hyperresponsiveness Thorax 2001 56 1 4 8 10.1136/thorax.56.1.4 11120896 PMC1745919

[B28] Luder E Melnik TA Dimaio M Association of being overweight with greater asthma symptoms in inner city black and Hispanic children Pediatrics 1998 132 699 703 10.1016/S0022-3476(98)70363-4 9580773

[B29] Romieu I Avenel V Leynaert B Kauffmann F Clavel-Chapelon F Body mass index, change in body silhouette, and risk of asthma in the E3N cohort study Am J Epidemiol 2003 158 2 165 74 10.1093/aje/kwg131 12851230

[B30] Nystad W Meyer HE Nafstad P Tverdal A Engeland A Body mass index in relation to adult asthma among 135,000 Norwegian men and women Am J Epidemiol 2004 160 10 969 76 10.1093/aje/kwh303 15522853

[B31] Young SN Gunzenhauser JD Malone KE Tiernan AM Body mass index and asthma in the military population of the northwestern United States Arch Intern Med 2001 161 1605 11 10.1001/archinte.161.13.1605 11434792

[B32] Cassol VE Rizzato TM Teche SP Basso DF Centenaro DF Maldonado M Moraes EZ Hirakata VN Solé D Menna-Barreto SS Obesity and its relationship with asthma prevalence and severity in adolescents from southern Brazil J Asthma 2006 43 1 57 60 10.1080/02770900500448597 16448967

[B33] Kaplan TA Montana E Exercise-induced bronchospasm in non-asthmatic obese children Clin Pediatr 1993 32 220 5 10.1177/000992289303200407 8462234

[B34] Del Río-Navarro B Cisneros-Rivero M Berber-Eslava A Espínola-Reyna G Sienra-Monge J Exercise induced bronchospasm in asthmatic and non-asthmatic obese children Allergol Immunopathol (Madr) 2000 28 1 5 11 PubMed PMID: 10757851 10757851

[B35] Tan RA Spector SL Exercise-induced asthma Sports Med 1998 25 1 6 10.2165/00007256-199825010-00001 9458523

[B36] Jarvis D Chinn S Potts J Burney P Association of body mass index with respiratory symptoms and atopy: results from the European Community Respiratory Health Survey Clin and Exp Allergy 2002 32 831 7 10.1046/j.1365-2222.2002.01380.x 12047427

[B37] Ford ES Mannino DM Redd SC Mokdad AH Mott JA Body mass index and asthma incidence among USA adults Eur Respir J 2004 24 5 740 4 10.1183/09031936.04.00088003 15516666

[B38] Beuther DA Sutherland ER A meta-analysis of prospective epidemiologic studies Am J Resp and Crit Care Med 2007 175 661 6 10.1164/rccm.200611-1717OC PMC1899288 17234901

[B39] Chen Y Dales R Krewski D Breithaupt K Increased effects of smoking and obesity on asthma among female Canadians. The National Population Health Survey 1994-1995 Am J Epidemiol 1995 15 255 62 10.1093/oxfordjournals.aje.a009996 10430229

[B40] Chen Y Dales R Tang M Krewski D Obesity may increase the incidence of asthma in women but not in men: longitudinal observation from the Canadian National Population Health Surveys Am J Epidemiol 2002 155 191 7 10.1093/aje/155.3.191 11821241

[B41] Huang SL Shiao G Chou P Association between body mass index and allergy in teenage girls in Taiwan Clin Exp Allergy 1999 29 323 9 10.1046/j.1365-2222.1999.00455.x 10202338

[B42] Chinn S Jarvis D Burney P European Community Respiratory Health Survey.Relation of bronchial responsiveness to body mass index in the ECRHS. European Community Respiratory Health Survey Thorax 2002 57 12 1028 33 10.1136/thorax.57.12.1028 12454296 PMC1758811

[B43] Chen Y Dales R Jiany Y The association between obesity and asthma is stronger in nonallergic than allergic adults Chest 2006 130 890 5 10.1378/chest.130.3.890 16963691

[B44] Grunstein RR Wilcox I Sleep-disordered breathing and obesity Baillieres Clin Endocrinol Metab 1994 8 601 28 10.1016/S0950-351X(05)80288-5 7980349

[B45] Gunnbjörnsdóttir MI Omenaas E Gíslason T Norrman E Olin AC Jõgi R Jensen EJ Lindberg E Björnsson E Franklin K Janson C Gulsvik A Laerum B Svanes C Torén K Tunsäter A Lillienberg L Gíslason D Blöndal T Björnsdottir US Jörundsdóttir KB Talvik R Forsberg B Franklin K Lundbäck B Söderberg M Ledin MC Boman G Norbäck D Wieslander G Spetz-Nyström U Cashelunge KS Rydén E RHINE Study Group. Obesity and nocturnal gastro-oesophageal reflux are related to onset of asthma and respiratory symptoms Eur Respir J 2004 24 1 116 21 15293613 10.1183/09031936.04.00042603

